# Pseudoginsenoside F11 Enhances the Viability of Random-Pattern Skin Flaps by Promoting TFEB Nuclear Translocation Through AMPK-mTOR Signal Pathway

**DOI:** 10.3389/fphar.2021.667524

**Published:** 2021-04-29

**Authors:** Feiya Zhou, Xian Zhang, Liangfu Jiang, Shi Li, Yiheng Chen, Jianbin Wu

**Affiliations:** ^1^ Department of Orthopaedics, The Second Affiliated Hospital and Yuying Children’s Hospital of Wenzhou Medical University, Wenzhou, China; ^2^ The Second Clinical Medical College of Wenzhou Medical University, Wenzhou, China

**Keywords:** random-pattern skin flaps, pseudoginsenoside F11, autophagy, angiogenesis, apoptosis, oxidative stress, TFEB, AMPK-mTOR signaling pathway

## Abstract

Random-pattern skin flap is widely used in tissue reconstruction. However, necrosis occurring in the distal part of the flap limits its clinical application to some extent. Activation of autophagy has been considered as an effective approach to enhance the survival of skin flaps. Pseudoginsenoside F11 (PF11), an ocotillol-type saponin, is an important component of Panax quinquefolium which has been shown to confer protection against cerebral ischemia and alleviate oxidative stress. However, it is currently unknown whether PF11 induces autophagy to improve the survival of skin flaps. In this study, we investigated the effects of PF11 on blood flow and tissue edema. The results of histological examination and western blotting showed that PF11 enhanced angiogenesis, alleviated apoptosis and oxidative stress, thereby improving the survival of the flap. Further experiments showed that PF11 promoted nuclear translocation of TFEB and by regulating the phosphorylation of AMPK. In summary, this study demonstrates that PF11 activates autophagy through the AMPK-TFEB signal pathway in skin flaps and it could be a promising strategy for enhancing flap viability.

## Introduction

Random-pattern skin flaps are commonly used in clinical practice to repair skin injury and cover wounds because of its high efficiency and convenience (Yu et al., 2013; Park et al., 2015). However, several post-operative complications limit its clinical application, the most serious of which was the flap necrosis (Ellabban et al., 2020). Lack of axial vasculature and insufficient oxygen supplement in the distal part of the flap are the main causes of necrosis (Namazi, 2014; Chang et al., 2017). Angiogenesis is a vital compensatory response to skin flap ischemia (Cury et al., 2013). After blood flow is restored, reperfusion of the affected tissues results in ischemia–reperfusion injury (IRI) also leads to flap necrosis (Wang W. Z. et al., 2011; Lin et al., 2017). This calls for the development of an effective method to increase the survival of skin flaps.

Autophagy is an important catabolic process that promotes cell survival by degrading cytosolic macromolecules and organelles (Betin and Lane, 2009; Bell et al., 2020). Various reports have shown that autophagy plays a cardinal role in regulating angiogenesis, apoptosis, and oxidative stress. For example, upregulated autophagy promotes angiogenesis and cardiac functional recovery after myocardial infarction (Liu et al., 2020a). Enhanced p53 degradation induces autophagy thereby attenuating oxidative stress-induced apoptosis (Liu et al., 2018). Moreover, in random-pattern skin flaps models augmented autophagy is conducive to enhance angiogenesis and reduce IRI (Lin et al., 2019; Zhou et al., 2019). Altogether, these findings imply that activation of autophagy may be an efficient strategy to improve the survival rate of skin flaps.

Pseudoginsenoside-F11 (PF11) is an ocotillol-type saponin found in *Panax quinquefolium* (Wang et al., 2013). PF11 has been reported to regulate macrophage polarization, and hence protect neurons from ischemic injury (Hou et al., 2020). In acute lung injury, PF11 was reported to suppress neutrophil infiltration and improve the neutrophil clearance rate (Wang P. et al., 2019). Moreover, animal models have shown that PF11 plays a protective role against Alzheimer’s disease and Parkinson’s disease (Song et al., 2017; Zhu et al., 2020). In the microglial cell model, PF11 was found to modulate TAK1/IKK/NF-κB and Akt signaling pathways to suppress the release of ROS and proinflammatory mediators (Wang et al., 2014). Interestingly, a recent study showed that PF11 upregulated autophagy and restored autophagy flow by promoting nuclear translocation of TFEB in neurons (Fu et al., 2021). However, the effect of PF11 on skin flaps has not been studied. Therefore, this study was designed to explore the effects of PF11-induced autophagy on random pattern skin flaps and the mechanisms involved.

## Materials and Methods

### Animals

A total of 132 healthy male Sprague Dawley rats (250–300 g) were obtained from Wenzhou Medical University (License No. SCXK [ZJ] 2005-0019). All rats received humane care and were housed in a room adjusted to 12 h light/dark cycles, humidity of 50 ± 5%, and temperature of 22–25°C. This study was approved by the Animal Care and Use Committee of Wenzhou Medical University (wydw 2017-0022) and conformed to the Guide for Care and Use of Laboratory.

### Reagents and Antibodies

The following reagents and antibodies were acquired as follows: PF11 (C_42_H_72_O_14_; purity ≥ 98%), 3MA (C_6_H_7_N_5_; purity ≥ 99.84%), and Compound C (CC, C_24_H_25_N_5_O; purity ≥ 99.65%) were obtained from MedChemExpress (Shanghai, China). The DAB developer, H&E Staining Kit, bovine serum, and pentobarbital sodium were purchased from Slarbio Science and Technology (Beijing, China). The 4,6-diamidino-2-phenylindole (DAPI), BCA Kit, RAPI Lysis Buffer, PMSF (Phenylmethanesulfonyl fluoride), Nuclear and Cytoplasmic Protein Extraction Kit (P0028), and the ECL Plus Reagent Kit (P0018FS) were bought from Beyotime Biotechnology (Jiangsu, China). Primary antibodies against α-SMA (14395-1), VEGF (19003-1), Superoxide Dismutase 1 (SOD1, 10269-1), Vacuolar Protein Sorting 34 (VPS34, 12452-1), Matrix Metalloproteinase (MMP9, 10375-2), Heme Oxygenase 1 (HO1, 10701-1), Cathepsin D (CTSD, 21327-1), Histone-H3(H3, 17168-1) and GAPDH (10494-1) were purchased from Proteintech Group (Chicago, IL, United States). The primary antibody against Cadherin 5 (A02632-2) was bought from Boster Biological Technology (Wuhan, China). Primary antibodies against AMPK-α (2532), phosphorylation-AMPK-α (p-AMPK-α, Ser485, 2537), mTOR (2983), phosphorylation-mTOR (p-mTOR, Ser2448, 2971), TSC2 (4308), phosphorylation-TSC2 (p-TSC2, Ser1387, 5584), phosphorylation-Raptor (p-Raptor, Ser792, 2083), Bcl-2 (3498), Bax (14796), Endothelial Nitric Oxide Synthase (eNOS, 32027), and Cleaved-caspase 3 (C-caspase3, 9661) were bought from Cell Signaling Technology (Beverly, MA, United States). The primary antibodies against SQSTM1/p62 (ab109012) and LC3 (ab192890) were obtained from Abcam (Cambridge, United Kingdom).

### Establishment of Animal Models Flaps

Rats were anesthetized with 3% pentobarbital sodium by intraperitoneal injection (60 mg/kg). After anesthesia, an additional dose was given during the surgery if necessary. Then the electric shaver was used to remove the dorsal fur. Subsequently, a skin flap (3 × 9 cm) was fixed on the back of the rat’s dorsum as previously described (Yu et al., 2013). The flap was covered on the donor bed and sutured to the original position using 4-0 non-absorbable sutures. The flap was evenly divided into three equal sections from the distal part to the pedicle: Area I, Area II, and Area III.

### Group Assignment and Treatment Protocols

A total of 132 rats were randomly divided into six groups: control group (*n* = 30), PF11 group (*n* = 30), PF11 + 3MA group (*n* = 24), PF11 + AAV-Negative control group (NC-shRNA, *n* = 18), PF11 + AAV-TFEB short hairpin RNA group (TFEB-shRNA, *n* = 18), and PF11 + CC group (*n* = 12). The PF11 group received PF11 (8 mg/kg/day, orally) for 7 days after creation of the flap model, while rats in the control group received an equal volume of saline using the same protocol. Rats in The PF11 + 3MA group were intraperitoneally injected with 3MA (10 mg/kg/day) 30 min before PF11 administration, given a total of 7°days. The PF11 + Compound C (CC) group received intraperitoneal injection of CC (1.5 mg/kg/day) 30 min before PF11 administration, for a total of 7°days. AAV-TFEB-shRNA (Genechem, Shanghai, China) was packed as previously reported. Fourteen days before flap surgery, the rats in PF11 + NC-shRNA group and PF11 + TFEB-shRNA group received daily subcutaneous injections of 18 micro ml viral vectors in PBS containing 5 × 10^9^ packaged genomic particles. After establishment of the flap model, rats in the two groups received PF11 as described above. Finally, all rats were euthanized by an overdose of pentobarbital sodium and tissue samples in area II were harvested for histological evaluation.

### Assessment of Flap Survival and Tissue Edema

On the 7th postoperation day (POD), the flap was photographed and then measured using Image-Pro Plus software (ver. 6.0; Media Cybemetics) and the percentage of viable area was calculated as: ×100%. The necrotic boundary was clear at the 7th POD, the distal part of the flap became harder and was covered with a dark nidus without new hair growth. On the contrary, the survival areas appeared pink, soft, and with new hair growth.

The water content was measured as an indicator of tissue edema in the flaps. On the 7th POD, flap specimens were dehydrated at 50°C. The specimen was weighed until the weight did not change anymore. The water content was calculated as previously described as [(wet weight − dry weight) ÷ wet weight] × 100% (Lin et al., 2018).

### Hematoxylin and Eosin Staining and Immunohistochemistry

On the 7th POD, six tissue samples from area II were fixed in paraformaldehyde and dehydrated. They were then embedded in paraffin wax and sectioned into 4°μm sections and stained with H&E using standard histology protocols. Finally, six random fields were observed for three sections under a light microscope (Olympus Corp, Tokyo, Japan). Microvascular density (MVD) was calculated as the number of micro-vessels per unit area (/mm^2^).

For the IHC assay, we performed as previously reported (Wu et al., 2019). In brief, the sections were first deparaffinized with xylene and rehydrated with ethanol (concentration: 100, 95, 85, and 75%). After that endogenous peroxidase was quenched with 3% H_2_O_2_ for 10 min and incubated with 10% bovine serum for 30 min at room temperature. Next, the sections were incubated with the primary antibodies: anti-Cadherin 5 (1:100), anti-CD34 (1:200), anti-CTSD (1:200), anti-SOD1 (1:200), and anti-C-caspase3 (1:200) at 4 C overnight. On the second day, the sections were washed three times with PBS and then incubated with HRP-conjugated secondary antibody and counterstained with hematoxylin. The sections were observed and images were captured under a light microscope (Olympus). The number of positive cells was counted using Image-Pro Plus software by observers who were blinded to the experimental groups.

### Immunofluorescence

As described above, six sections were deparaffinized and rehydrated. After three times washing and tissue antigen was retrieved with sodium citrate buffer at 95 C for 20 min. Next, 0.1% (v/v) PBS-Triton X-100 was used to permeabilize the samples. The slides were blocked in 10% (v/v) goat serum albumin dissolved in PBS for 1°h at room temperature. Finally, sections were incubated with primary antibody against α-SMA (1:200), LC3 (1:200), and TFEB (1:100) at 4 C overnight. This was followed by incubation with FITC-conjugated secondary antibody at room temperature for 1 h. The nuclei were counterstained with DAPI. Images of each slide were captured randomly under a fluorescent microscope (Olympus Inc., Tokyo, Japan). The number of positive cells was analyzed using Image-Pro Plus software.

### Laser Doppler Perfusion Image

Six rats in each group were placed on a prone position under anesthesia at room temperature. The Laser Doppler instrument (Moor Instruments, Axminister, United Kingdom) was used to scan the full field of the flap to assess the blood supply in the flaps on the 7th POD. Blood flow was quantified and analyzed using the Moor LDI Review software (ver.6.1; Moor Instruments). Each rat was scanned three times and the mean value was calculated.

### Western Blotting Analysis

Six tissue samples in each group were harvested from the middle part of area II for western blotting analysis. The tissues were homogenized with lysis buffer containing RIPA and 1 mmol/L PMSF and after centrifugation, the total proteins were gathered. The Nuclear and Cytoplasmic Protein Extraction Kit was used to separate cytoplasmic and nuclear protein. First, cut the tissue into small pieces and homogenized it with cytoplasmic protein extraction reagents A and B on the ice. After 5 min centrifugation at 1,500 rpm and the supernatant is the cytoplasmic protein. Second, add the nuclear protein extraction reagents and vortex on the highest setting for 15 ss and continue vortexing for 15 ss every 2 min, for a total of 30 min. After 10°min centrifugation at 13,000 rpm and transfer the supernatant (nuclear extract) to a clean pre-chilled tube. Finally, the protein concentration in the samples was determined by the BCA assay kit. The proteins were resolved on 12% (w/v) gel and transferred onto PVDF membranes. After blocking with 5% skimmed milk for 120°min, the membranes were incubated with the corresponding primary antibodies overnight at 4 C. The membranes were subsequently incubated with a secondary antibody for 2 h at room temperature. Finally, the ECL Plus Reagent Kit was used to visualize the protein bands and the gray value of the band was analyzed using Image Lab 3.0 (Bio-Rad).

### Statistical Analyses

Statistical analyses were performed using SPSS 22 software (Chicago, IL, United States). All data are presented as mean ± standard deviation (SD). Differences among the groups were compared with one-way ANOVA or *t*-test. *p*-values less than 0.05 were considered statistically significant.

## Results

### PF11 Promotes the Survival of Random Skin Flaps

**FIGURE 1:**
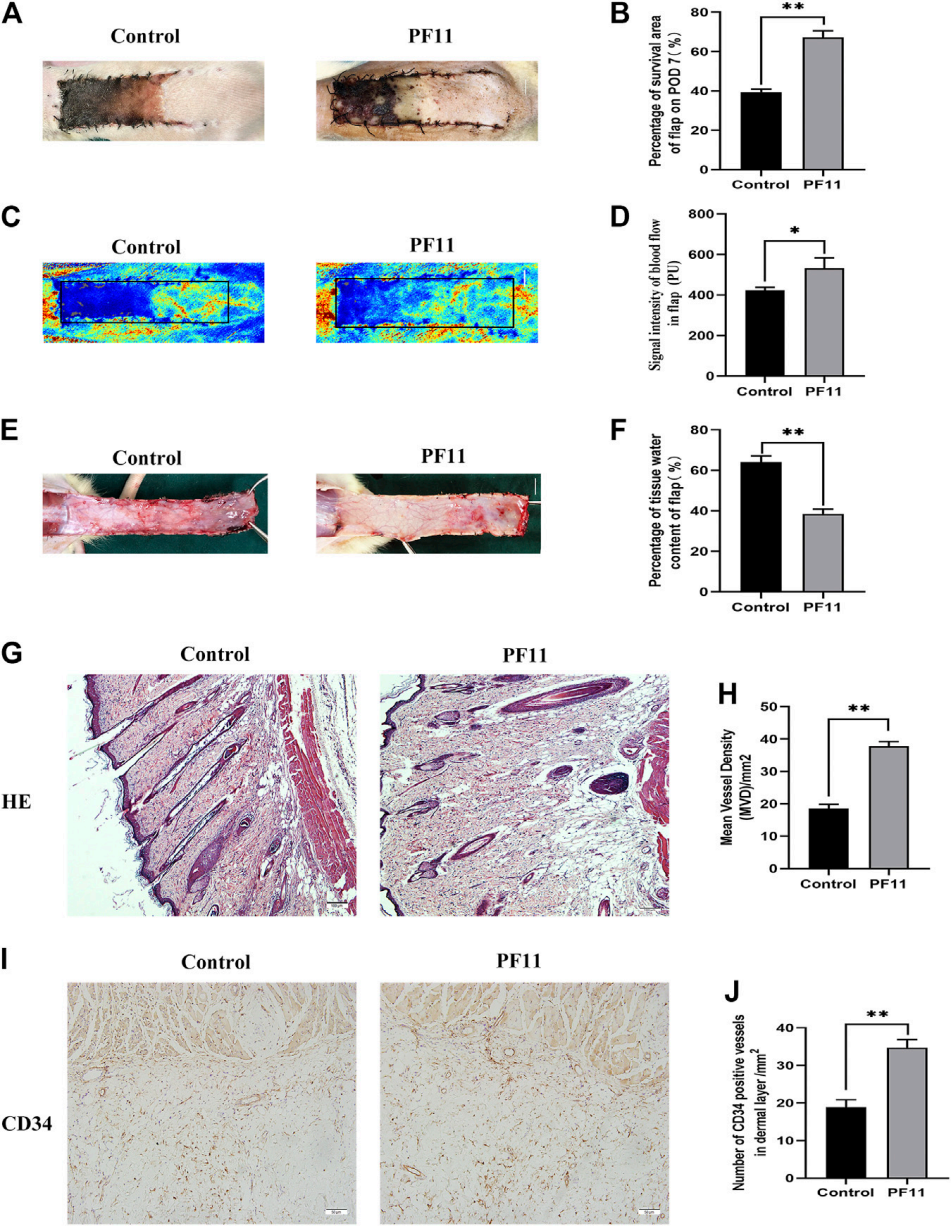
**(A)** Digital photographs of flap on the 7th POD (scale bar, 1 cm). **(B)** The histogram showing the percentage of survival area of the flap on the 7th POD. **(C)** Laser Doppler images showing vascular network and blood supply on the 7th POD in the control and PF11 groups (scale bar, 1 cm). **(D)** Histogram showing the signal intensity of blood flow. **(E)** Photograph showing edema on the inner side of the skin flap (scale bar, 1 cm). **(F)** Histogram showing the percentage of tissue water content **(G,H)** H&E staining showing MVD in Area II of flaps in the control and PF11 groups (original magnification ×100; scan bar, 100 μm). **(I,J)** IHC results showing the density of CD34 labeled vessels (/mm^2^). Values are expressed as means ± SD, *n* = 6 per Group. *p < 0.05 and **p < 0.01, vs. control group.

Necrosis gradually developed in the flap from the distal part to the pedicle, and the tissue became stiff, dark, crumpled, and without new hair growth (Figure 1A). The survival area in PF11 group was larger than that in the control group on the7th POD (Figure 1B). Then Laser Doppler visualized the blood flow in the flap and results are shown in (Figure 1C). The PF11 group exhibited a stronger blood flow signal value than the control group on 7th POD (Figure 1D). Moreover, Flaps with venous blood stasis and tissue edema were more significant in the control group compared to the PF11 group (Figure 1E). The water content in the flaps was lower in PF11 group than in the control group (Figure 1F). Besides, H&E staining results showed that the microvascular density of PF11 group was significantly higher compared with that of control group (Figures 1G,H). Likewise, CD34 labeled vessels were much more in the PF11 group (Figures 1I,J). Collectively, these results demonstrated that PF11 promoted the survival of flaps.

### PF11 Enhances Angiogenesis in Skin Flaps

**FIGURE 2:**
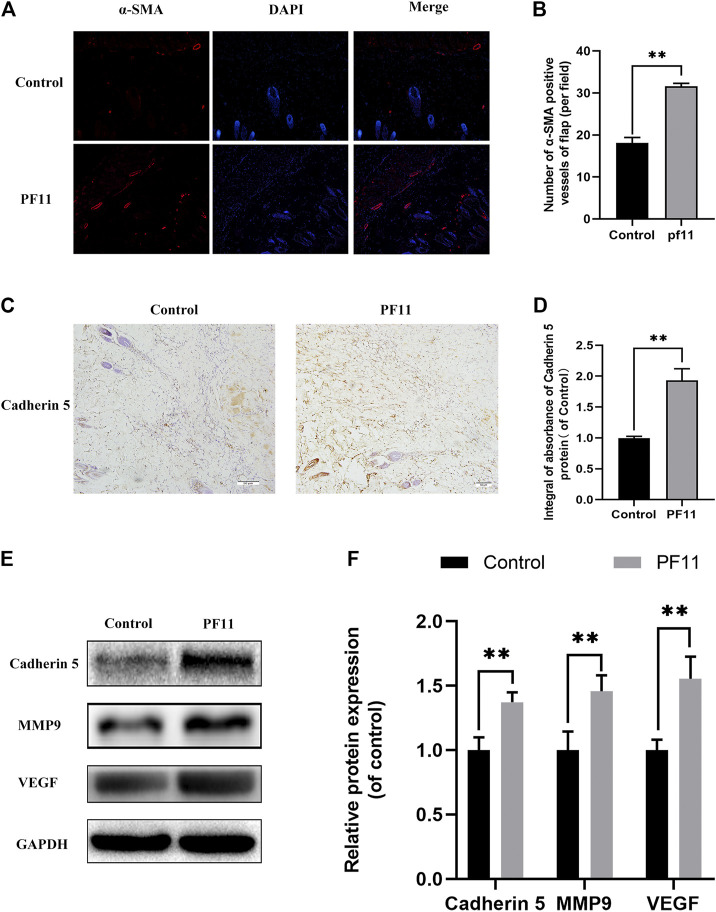
PF11 enhances angiogenesis in random skin flaps. **(A)** Immunofluorescence staining for α-SMA labeled microvessels (scale bar: 20 µm). **(B)** Histogram showing the percentages of a-SMA positive microvessels in the control and PF11 groups. **(C)** IHC results showing the Cadherin 5 expression in random skin flaps (scan bar, 50 μm). **(D)** A histogram exhibiting the integral absorbance of Cadherin 5. **(E)** Results of western blotting showing the expression of MMP9, VEGF, Cadherin 5, and GAPDH. Cropped blots are shown. **(F)** Histogram showing optical density values of MMP9, VEGF, and Cadherin5 in the control and PF11 group in the flaps. Values are expressed as means ± SD, *n* = 6 per Group. *p < 0.05 and **p < 0.01, vs. control group.

The protein α-SMA is primarily expressed in vascular smooth muscle cells. Immunofluorescence showed that the number of α-SMA positive microvessels in the PF11 group was much more than that in the control group (Figures 2A,B). The expression of proteins associated with angiogenesis was determined using IHC and western blotting in the skin flaps. Results from both assays showed that the protein level of Cadherin 5 was significantly higher in the PF11 group compared to the control group (Figures 2C–F). Moreover, PF11 treatment increased the expression level of VEGF and MMP9 which was important in the angiogenesis process (Figures 2E,F). Altogether, these findings indicated that PF11 promoted angiogenesis.

### PF11 Reduces Oxidative Stress and Alleviates Apoptosis in Flaps

**FIGURE 3:**
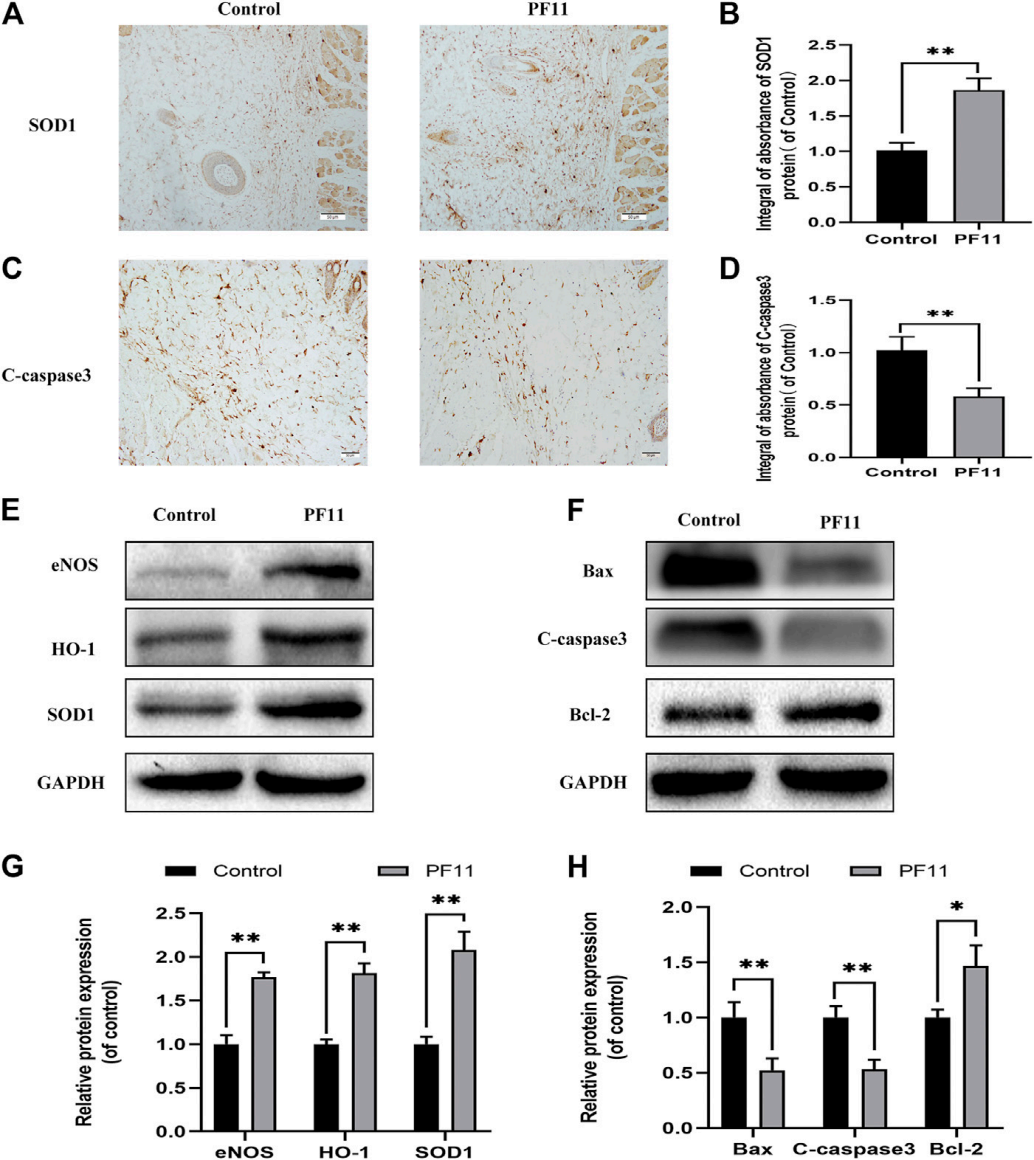
PF11 reduces oxidative stress and alleviates apoptosis in skin flaps. **(A)** IHC was performed to assess the level of SOD1 positive cells in skin flaps (scan bar, 50 μm). **(B)** The integral absorbance of SOD1 in the dermal layer. **(C)** The expression of C-caspase3 was evaluated by IHC in Area II of skin flaps (scan bar, 50 μm). **(D)** The integral absorbance of C-caspase3 was quantified by Image-Pro Plus. **(E,F)** Results of western blotting showing the expression of SOD1, eNOS, HO1, Bax, Bcl-2, and C-caspase3. Cropped blots are shown. **(G,H)** The optical density values of SOD1, eNOS, HO1, Bax, Bcl-2, and C-caspase3 expression in the flaps. Values are expressed as means ± SD, *n* = 6 per Group. *p < 0.05 and **p < 0.01, vs. control group.

Oxidative stress plays an important role during the reperfusion period in skin flaps. Therefore, IHC and western blotting assays were carried out to determine the expression of SOD1 in skin flaps (Figures 3A,E). Results of both IHC and western blotting assays showed that SOD1 expression was higher in PF11 group than in control group (Figures 3B,G. eNOS has antioxidant activity during oxidative stress (Yu and Liu, 2018). HO-1 is well known as a downstream target protein of Nrf2 and has a strong antioxidant capacity (Liu et al., 2020b). Further analysis showed that PF11 treatment increased eNOS and HO1 expression (Figures 3E,G). These results showed that PF11 decreased the oxidative stress level in flaps.

Apoptosis is one of the causes of flap necrosis. Therefore, we asked whether PF11 alleviates apoptosis in skin flaps. First, IHC was carried out to assess and examine the expression of a marker of apoptosis, C-caspase 3. We found that the PF11 group had a lower level of C-caspase 3 than the control group (Figures 3C,D). Western blotting assay showed that PF11 treatment downregulated the levels of C-caspase 3 and Bax (Figures 3F,H). Furthermore, PF11 upregulated the expression of Bcl-2, an anti-apoptosis protein (Figures 3F,H). Altogether, these results suggested that PF11 inhibited cell death by reducing apoptosis.

### PF11 Increased Autophagy Flux in Random Skin Flaps

**FIGURE 4:**
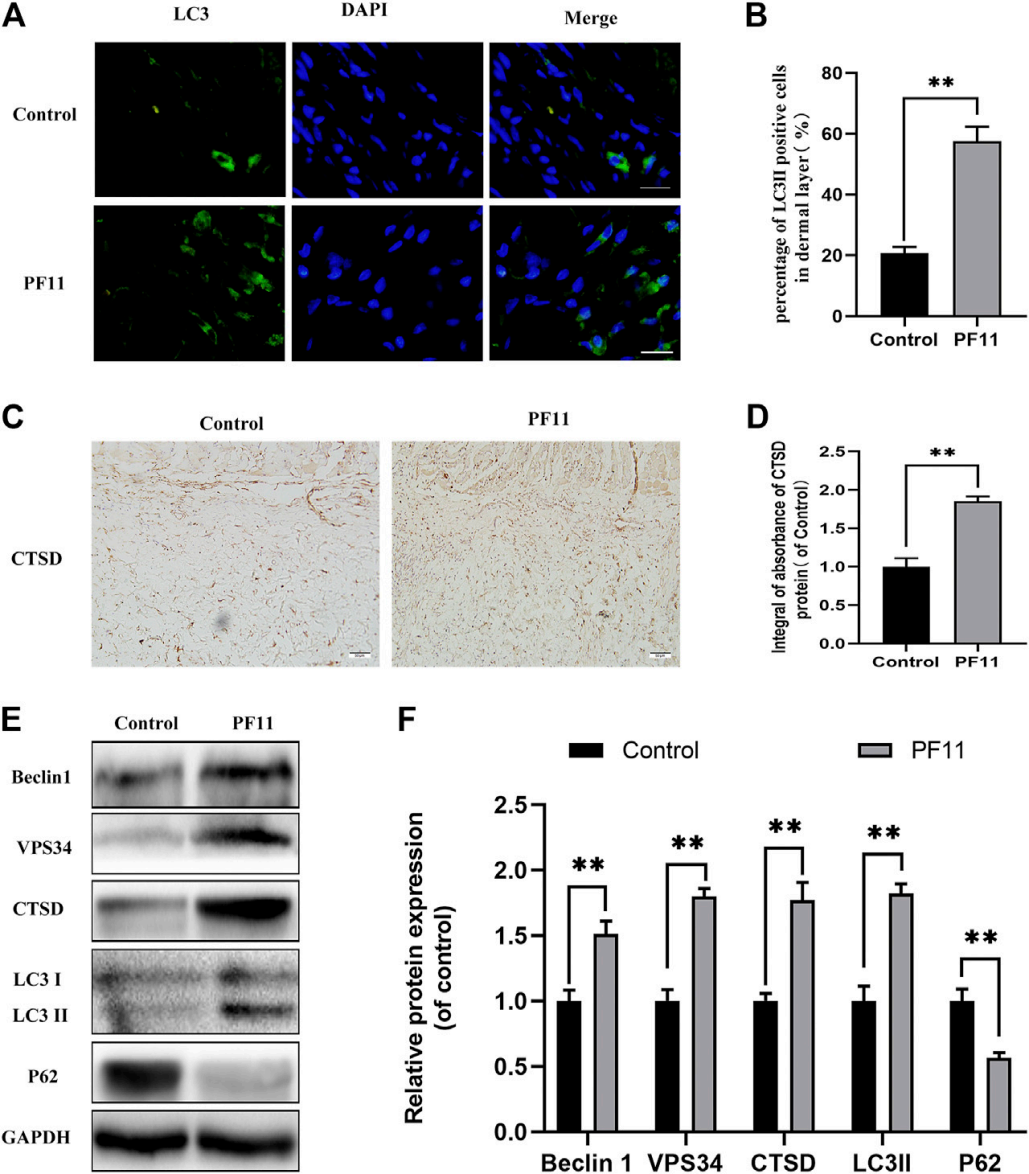
PF11 increased autophagy flux in random skin flaps. **(A)** Results of immunofluorescence staining for LC3II positive cells in the dermal layer showing the autophagosomes (red) in cells in Area II of flaps (scale bar: 20 μm). **(B)** Histogram showing the percentages of LC3II positive cells. **(C)** IHC results showing the level of CTSD in the control and PF11 groups (scan bar, 50 μm). **(D)** The integral absorbance of CTSD protein in the dermal layer. **(E,F)** Results of western blotting showing the expression of Beclin1, VPS34, CTSD, p62, and LC3II. Values are expressed as means ± SD, *n* = 6 per Group. *p < 0.05 and **p < 0.01, vs. control group.

To investigate whether PF11 activates autophagy in flaps, the expression of Beclin1, VPS34, LC3II, p62, and CTSD was quantified. Beclin1, VPS34, and LC3II are markers of autophagosome formation (Parzych and Klionsky, 2014). CTSD is a lysosomal acid protease and is involved in proteolytic degradation (Medina et al., 2015). p62 is a marker for autophagy flux degraded during the autophagic process (Hill et al., 2021). Results of immunofluorescence staining showed that the number of LC3II-positive cells in the PF11 group was higher than that in the control group (Figures 4A,B). Moreover, IHC analysis revealed that PF11 enhanced the expression of CTSD (Figures 4C,D). Finally, western blotting was performed to measure the expression of Beclin1, VPS34, LC3II, p62, and CTSD. The results revealed that PF11 group had higher levels of CTSD, VPS34, Beclin1, and LC3II but a lower level of p62 compared to control group (Figures 4E,F). The results revealed that the PF11 group had higher levels of VPS34, Beclin1, and LC3II suggesting that more autophagosomes were formed than in the control group. Moreover, compared with the control group the level of P62 was decreased but CTSD expression was elevated in the PF11 group which indicated that autophagic flux was progressing smoothly. In conclusion, these findings showed that PF11 increased autophagy flux in random skin flaps.

### 3MA Inhibits Autophagy and Reverses the Positive Effects of PF11 on Skin Flaps

**FIGURE 5:**
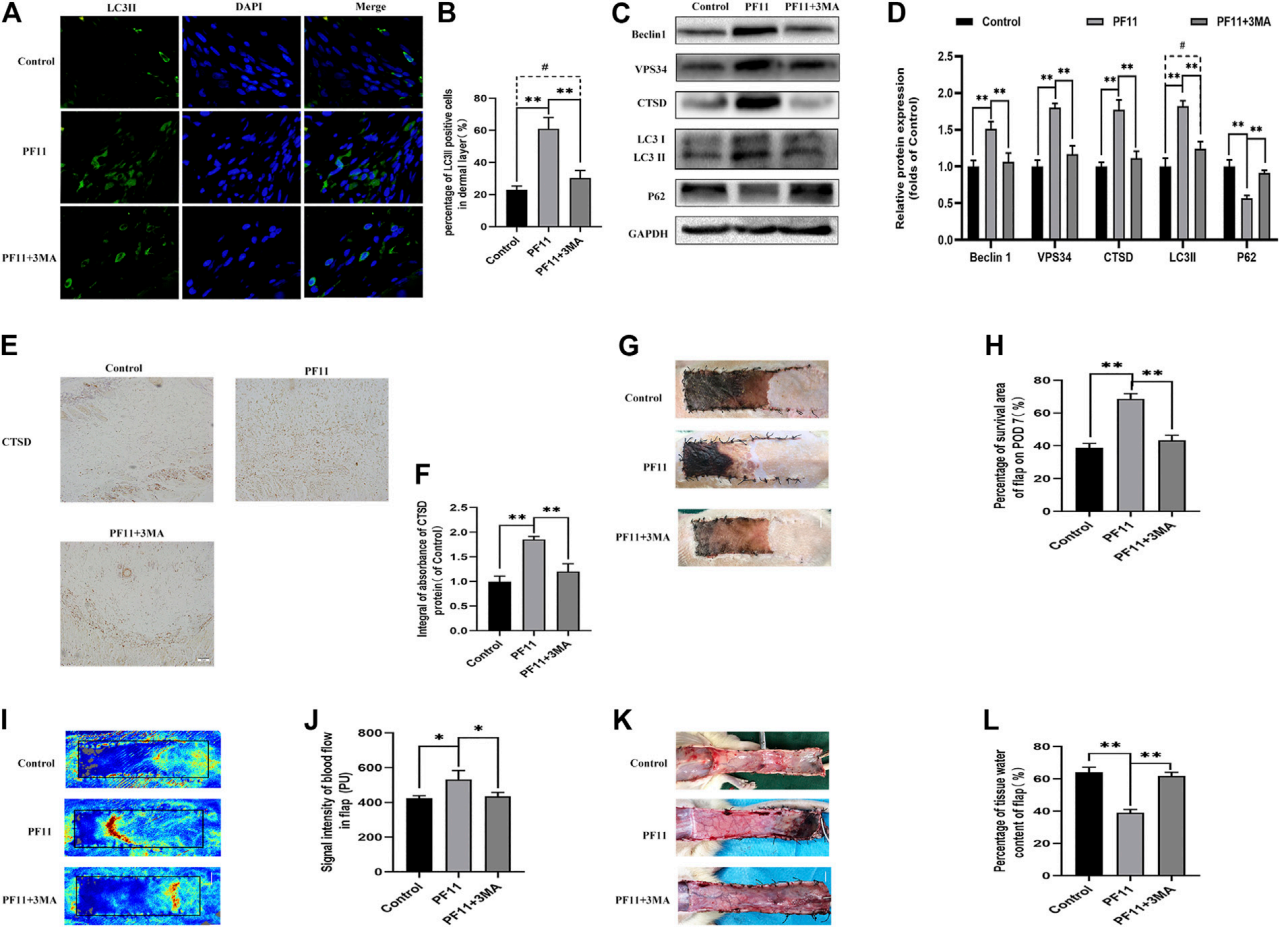
Inhibition of autophagy reverses the positive effects of PF11 on random-pattern skin flap. **(A,B)** Immunofluorescence staining for LC3II positive cells in Area II of flaps (scale bar: 20 μm). **(B)** A histogram showing the percentage of LC3II positive cells. **(C,D)** Western blotting was performed to assess the expression of autophagy-related protein Beclin1, VPS34, CTSD, p62, and LC3II. **(E,F)** IHC results showing the expression of CTSD in the control, PF11, and PF11 + 3MA groups (scan bar, 50 μm). **(G,H)** Digital photographs showing the percentage of survival area of the flap on the 7th POD (scale bar, 1 cm). **(I,J)** The signal intensity of blood flow as detected by Laser Doppler on the 7th POD (scale bar, 1 cm). **(K,L)** Photograph exhibiting edema and vascular network on the inner side of the skin flap (scale bar, 1 cm). Values are expressed as means ± SD, *n* = 6 per group. *p < 0.05 and **p < 0.01, vs. PF11 group. #*p* < 0.05 and ##*p* < 0.01, vs. control group.

A previous study reported that augmented autophagy in random-pattern skin flaps models is conducive to enhance angiogenesis and reduce IRI (Liu et al., 2017). To determine whether the effect of PF11 on flap survival is mediated by upregulation of autophagy, we co-administrated 3MA, an autophagy inhibitor, with PF11. The results of immunofluorescence showed that among three groups the number of LC3II-positive cells was highest in PF11 group and lowest in control group (Figures 5A,B). Then, IHC analysis revealed that the expression of CTSD was declined in the PF11 + 3MA group (Figures 5E,F). Furthermore, western blotting results demonstrated that 3MA co-treatment decreased the levels of LC3 II, Beclin1, VPS34, and CTSD but increased the expression of p62, while there was no significant difference between control and PF11 + 3MA group (Figures 5C,D). Together, these data indicated that 3MA effectively inhibited the PF11 induced autophagy.

**FIGURE 6:**
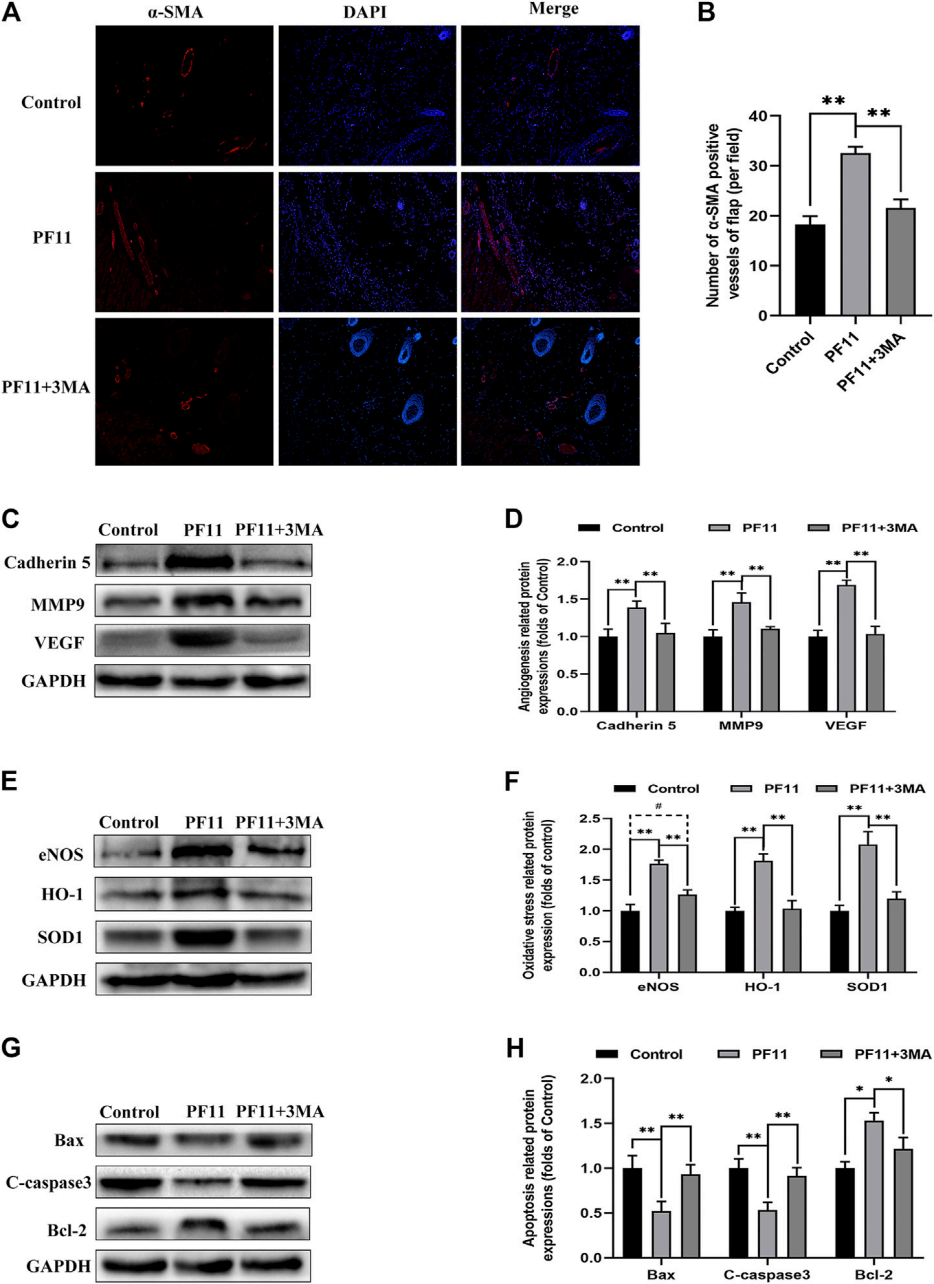
Inhibition of autophagy abolished the positive effects of PF11 on angiogenesis, apoptosis, oxidative stress in skin flaps. **(A,B)** Immunofluorescence staining showed the α-SMA positive microvessels in the control, PF11, and PF11 + 3MA groups (scale bar: 20 µm). **(C,D)** Western blotting results exhibited the expression of angiogenesis-related protein MMP9, VEGF, and Cadherin 5. **(E,F)** Western blotting results exhibited the expression of oxidative stress-related protein SOD1, HO1, and eNOS in the control, PF11, and PF11 + 3MA groups. **(G,H)** Western blotting results exhibited the expression of apoptosis-related protein Bax, Bcl-2, and C-caspase3. Values are expressed as means ± SD, *n* = 6 per group. *p < 0.05 and **p < 0.01, vs. PF11 group. #*p* < 0.05 and ##*p* < 0.01, vs. control group. #*p* < 0.05 and ##*p* < 0.01, vs. control group.

Next, we examined some indicators of flap viability in PF11 group and PF11 +3MA group. In the beginning, digital photographs revealed the survival/necrosis area. Qualitatively, we found that the flap survival rate was lower in PF11 + 3MA group than in the PF11 group; meanwhile, the survival area was larger in PF11 + 3MA group than in the control group though without significance (Figures 5G,H). Besides, co-treatment with 3MA significantly decreased the blood flow signal as evidenced from the Laser Doppler results on 7th POD (Figures 5I,J). Moreover, co-treatment with 3MA aggravated tissue edema reversed the effect of PF11 on flaps (Figures 5K,L). At the molecular level, immunofluorescence showed that the number of were decreased after co-administrated with 3MA compared with PF11 group while the α-SMA positive microvessels numbers were higher in the 3MA group than the Control group which means that 3MA treatment partly suppressed PF11’s angiogenesis effects (Figures 6A,B). Likewise, the western blotting analysis further showed that the angiogenesis-related proteins MMP9, VEGF, and Cadherin 5 were significantly reduced in PF11 + 3MA group compared with the PF11 group and the difference between the PF11 + 3MA group and control group was not statistically significant (Figures 6C,D), indicating that the pro-angiogenesis effect of PF11 was mediated by autophagy in the flap model. Similarly, 3MA significantly decreased the expression of anti-oxidative stress-related proteins (SOD1, eNOS, and HO1), though the expression of eNOS was still higher in the PF11 + 3MA group than in the control group, demonstrating that PF11 induced autophagy alleviated oxidative stress in skin flaps (Figures 6E,F). Finally, the results of Bax, Bcl-2, and C-caspase3 expression further indicated that 3MA blocked the effects of PF11 on apoptosis (Figures 6G,H). To sum up, these results showed that inhibition of autophagy largely abolished the benefits of PF11 on skin flaps.

### PF11 Augments Autophagy Flux by Promoting TFEB Nuclear Translocation

**FIGURE 7:**
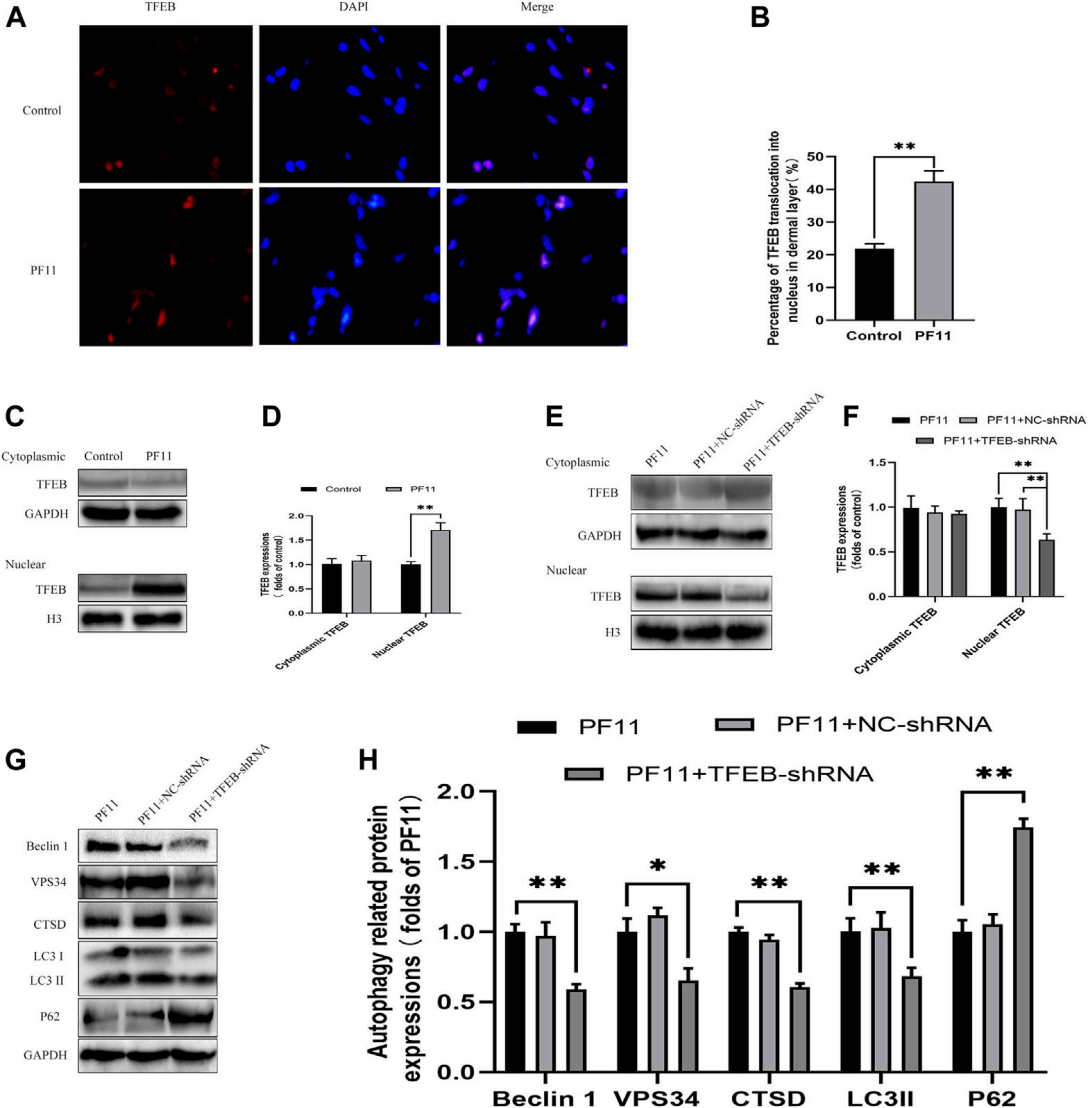
PF11 augments autophagy by promoting nuclear translocation of TFEB. **(A,B)** Immunofluorescence staining for nuclear translocation of TFEB (red) in cells of flaps in the control and PF11 groups (scale bar, 20 μm). **(C,D)** Western blotting was performed to assess the expression of cytoplasmic and nuclear TFEB in the control and PF11 group. **(E,F)** The level of cytoplasmic and nuclear TFEB in the PF11, PF11 + NC-shRNA, and PF11 + TFEB-shRNA groups were evaluated by western blotting assay **(G,H)** Western blotting was used to assess the expression level of p62, Beclin1, VPS34, CTSD, and LC3II in the PF11, PF11 + NC-shRNA, and PF11 + TFEB-shRNA groups. Cropped blots are shown. Values are expressed as means ± SD, *n* = 6 per group. *p < 0.05 and **p < 0.01, vs. PF11 group.

Many studies have reported that TFEB regulates autophagy (Pastore et al., 2020). Here, we determined whether PF11 modulates autophagy via enhancing TFEB expression in skin flaps. Qualitative analysis of immunofluorescence images showed that the PF11 group had much stronger nuclear translocation of TFEB than the control group (Figures 7A,B). Then western blotting results of the expressions of cytoplasmic TFEB and nuclear TFEB also verified the Immunofluorescence results revealed that the level of nuclear TFEB was upregulated by PF11 treatment (Figures 7C,D).

To further certified that PF11-induced autophagy was mediated by upregulating TFEB, we injected the TFEB-shRNA AAV vector to reduce TFEB activity and blank AAV vector as a negative control. Western blotting results showed the levels of nuclear TFEB were lower in PF11 + TFEB -shRNA group compared to PF11 and PF11 + NC-shRNA groups. However, the expression difference of cytoplasmic TFEB was not statistically significant. (Figures 7E,F). This implies that TFEB -shRNA effectively inhibited TFEB expression in skin flaps. Besides, the level of autophagosome-related proteins VPS34, Beclin1, LC3II, and lysosomal-related protein CTSD was significantly lower, whereas that of autophagy flux marker p62 was higher in the TRE + TFEB-shRNA group compared to the PF11 and PF11 + NC- shRNA groups (Figures 7G,H). Notably, the expression of these proteins was not significantly different between PF11 and PF11 + NC-shRNA groups (Figures 7G,H). These data suggested that degradation of autophagosomes was blocked after TFEB was knocked down. Therefore, we could conclude that PF11may indeed regulate autophagy flux by promoting TFEB nuclear translocation in random-pattern skin flaps.

### PF11 Regulates the AMPK-mTOR Pathway in Flaps

**FIGURE 8:**
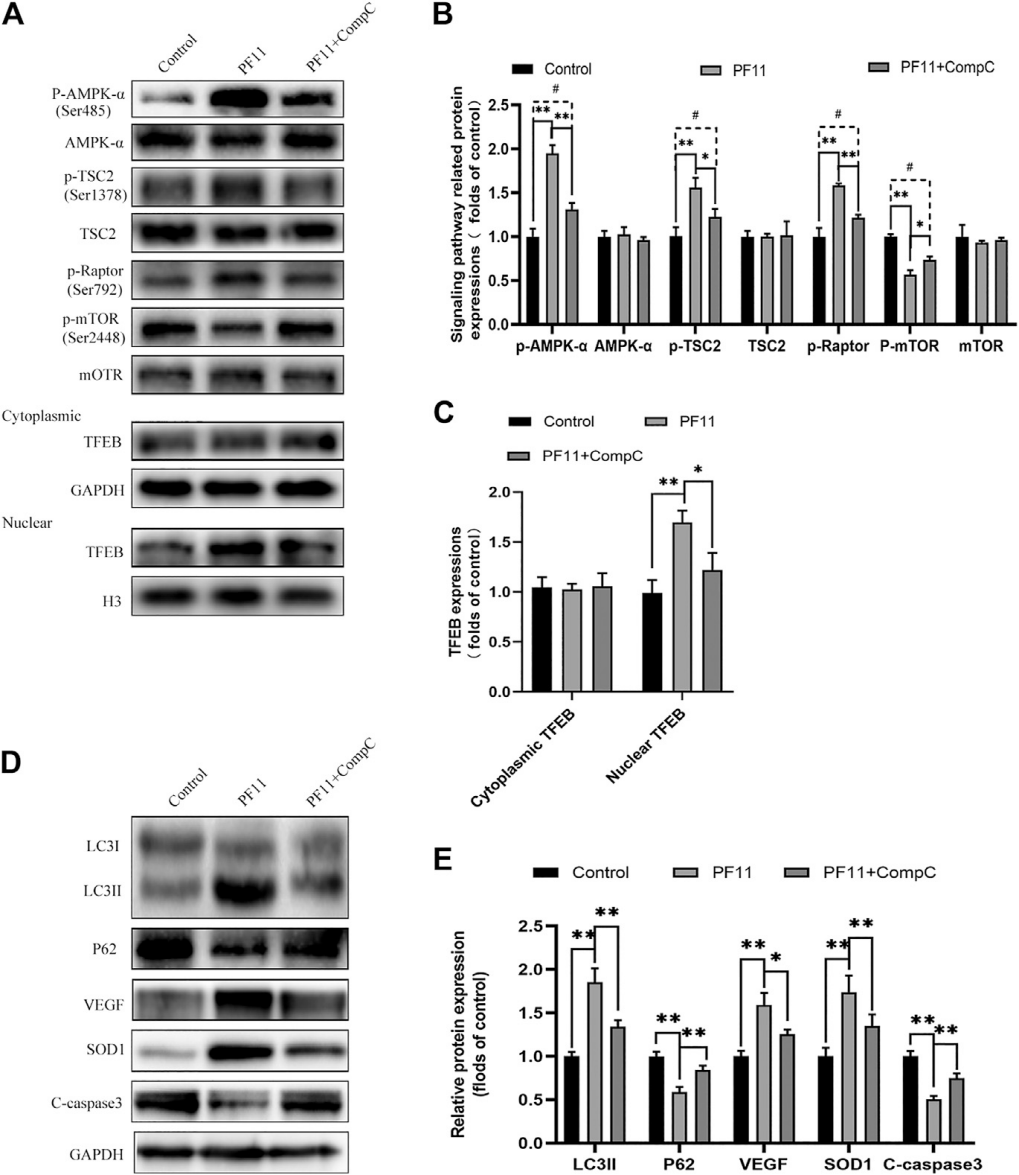
PF11 regulates AMPK-mTOR signaling pathway in flaps. **(A–C)** Western blotting results showed the expression of AMPK-α, p-AMPK-α, TSC2, p-TSC2, p-Raptor mTOR, p-mTOR, cytoplasmic TFEB, and nuclear TFEB in the control, PF11, and PF11 + CC groups. **(D,E)** Expression and quantification of LC3II, p62, VEGF, SOD1, and C-caspase3 in each group. Values are expressed as means ± SD, *n* = 6 per group. *p < 0.05 and **p < 0.01, vs. PF11 group. #*p* < 0.05 and ##*p* < 0.01, vs. control group.

Past studies have revealed that activation of the AMPK-mTOR pathway can modulate TFEB nuclear translocation (Bruiners et al., 2020). To further explore the mechanisms by which PF11 affected the nuclear translocation of TFEB in the flap, we examined the AMPK-mTOR signaling pathway. Compound C, an AMPK blocker, was applied to inhibit AMPK phosphorylation. Western blotting results indicated that the expression of p-AMPK and the nuclear translocation of TFEB were upregulated in PF11-treatment group but were decreased by compound C (Figures 8A–C). To identify that p-mTOR (Ser2448) is dephosphorylated via AMPK and not PI3 kinase/Akt activation, the TSC2 at Ser-1387 and Raptor at Ser-792 was detected by western blot. The TSC2, a GTPase activating protein, is a major input from AMPK into the mTORC1 pathway. Knockdown TSC2 can reverse mTORC1 inhibition upon AMPK activation (Inoki et al., 2003). Raptor is a binding protein in the mTORC1 complex, where it regulates the complex activity. AMPK can phosphorylate Raptor (Ser-792) and lead to the inhibition of mTORC1 (Hara et al., 2002; Kim et al., 2002). As shown in Figures 8A,B the expression level of p-TSC2 (Ser-1387) and p-Raptor (Ser-792) were increased in PF11 group but were decreased in PF11 + Comp C group. We also measured the level of p-mTOR (Ser2448) and found that PF11 inhibited mTOR activation and compound C increased the expression level of p-mTOR (Figures 8A,B). Of note, the expression level of AMPK, TSC2, mTOR, and cytoplasmic TFEB was not different between PF11-treated and compound C -treated groups (Figures 8A–C). Finally, treatment with compound C decreased the expression of Beclin1, LC3II, VPS34, CTSD, and increased the expression of p62, indicating that compound C inhibited PF11-induced autophagy (Figures 8D,E). Overall, these results demonstrated that PF11 promoted nuclear translocation of TFEB by regulating the AMPK-mTOR pathway in skin flaps.

## Discussion

PF11 is a natural compound extracted from the roots and leaves of Panax quinquefolium (American ginseng) (Wang Z.-J. et al., 2011; Zhu et al., 2020), It has been reported to confer therapeutic effects in many diseases. Studies involving cellular and animal models have reported that PF11 antagonizes the behavioral effects of morphine in mice (Li et al., 2000), against oxidative stress-induced damage (Wang et al., 2013), and enhances neuronal activity (Wang Z.-J. et al., 2011). We, therefore, hypothesized that PF11 may improve the survival of skin flaps. Our current work showed that PF11 promotes the survival of random skin flaps by augmenting autophagy, neovascularization, and alleviating oxidative stress and apoptosis. Furthermore, PF11 induced autophagy by increasing the nuclear translocation of TFEB through the AMPK-mTOR signaling pathway in random skin flaps.

Angiogenesis involves attachment, matrix degradation, migration, and proliferation processes, which are regulated by multiple cytokines and signaling pathways (Lin et al., 2014; Xie and Berger, 2019). VEGF influences the mitosis of vascular, stimulates the proliferation of vascular endothelial cells and fibroblasts (Shaw and Collen, 2000). MMP9 stimulates the migration of endothelial cells and angiogenesis (Xu et al., 2001). Cadherin 5, on the other hand, promotes the formation during angiogenesis (Sasaki et al., 2020). The results showed that VEGF, MMP9, and Cadherin 5 protein levels were higher in PF11 group compared with control group. Altogether, our data indicated that PF11 improved the survival of skin flaps partly by promoting angiogenesis.

After neovascularization and partial restoration of the blood supply, IRI ensues. IRI disrupts capillaries leading to tissue edema (Molski et al., 2009; Karasawa et al., 2015). Aggregation of reactive oxygen species (ROS) triggers flap necrosis (Storz and Imlay, 1999). Studies have reported that PF11 treatment alleviates cognitive impairment by suppressing oxidative stress in d-galactose-treated mice (Zhang et al., 2019). In our study, we observed that PF11 increased the levels of anti-oxidative stress related proteins SOD1, HO1, and eNOS in the random skin flaps implying that PF11 has anti-oxidative stress effects. Reperfusion triggers the overproduction of ROS which may lead to mitochondrial dysfunction and apoptosis (Daverey and Agrawal, 2020; Huang et al., 2020). Inhibition of apoptosis effectively prevents cell death. Therefore, we measured apoptosis-related proteins. Bcl-2 is a well-known anti-apoptosis protein that regulates mitochondrial outer membrane permeabilization. Bax and C-caspase3 also regulate apoptosis. In the present work, we found that PF11 upregulated Bcl-2 expression and downregulated Bax and C-caspase3 expression. These data suggested that PF11 enhanced flap survival by alleviating IRI-triggered oxidative stress and inhibiting apoptosis.

Autophagy degrades intracellular waste materials during nutrient deficiency (Matsui et al., 2007). Autophagy regulates tissue homeostasis (Takagi et al., 2007), and previously showed that activation of autophagy improved the survival of random-pattern skin flaps (Yuan et al., 2019). PF11 exerted neuroprotective effects during stroke by augmenting autophagy and restoring autophagy flux (Yuan et al., 2020). However, there is no study has investigated the effect of PF11 on autophagy in skin flaps. We, therefore, assessed the markers of the autophagy-lysosome pathway, LC3, Beclin1, VPS34, CTSD, and P62, to determine the effect of PF11 on autophagy (Fang et al., 2020). The results revealed that VPS34, Beclin 1, LC3II, and CTSD were higher in PF11 group compared to the control group indicated that PF11 may not only promote autophagosome formation but also elevated the autophagic flux. Furthermore, the lower expression of P62 in PF11 group illustrated that the progress of autophagic degradation was progressing smoothly. In contrast, treatment with 3MA produces opposite results. Moreover, treatments with 3MA abolished the pro-angiogenesis, anti-oxidative stress, and anti-apoptotic of PF11. These results demonstrated that PF11 improved skin flap survival by regulating autophagy flux.

Evidence indicates that TFEB is a novel positive regulator of autophagy and lysosome biogenesis (Pastore et al., 2019; Wang Y.-T. et al., 2019). Activation of TFEB with pharmacological agents has been shown to confer beneficial effects in various cellular and animal disease models. For example, TFEB improved the progression of Parkinson’s disease and liver steatosis. (Ren et al., 2019; Wang et al., 2020). TFEB also regulates angiogenesis by activating AMPKα and autophagy in endothelial cells (Fan et al., 2018). Recently, PF11 was shown to significantly alleviate autophagy flux defects in a cerebral ischemia injury model by increasing the nuclear translocation of TFEB (Fu et al., 2021). In our study, we found that PF11 enhanced the nuclear translocation of TFEB in skin flaps. To further investigate whether TFEB was involved in the PF11-induced autophagy, we inhibited TFEB translocation using AAV-TFEB-shRNA. The results showed that autophagy level was downregulated in PF11 + TFEB-shRNA group and the therapeutic benefits of PF11 on flap survival were suppressed. Altogether, these findings indicated that PF11 upregulated autophagy by enhancing nuclear translocation of TFEB, thereby promoting the survival of random skin flaps.

Further experiments showed that the AMPK signaling pathway was affected by PF11 treatment. AMPK serves as a fuel-sensing enzyme in eukaryotic cells (Salt and Hardie, 2017). Increased AMP to ATP ratio can activate AMPK (Zhang et al., 2017). Activation of AMPK promotes angiogenesis and increases the supply of nutrients (Zhu et al., 2019). Moreover, some studies have shown that the AMPK signaling pathway plays a cardinal role in activating TFEB (Collodet et al., 2019). Furthermore, regulation of the AMPK-mTOR signaling pathway induces dephosphorylation of TFEB and allows TFEB to translocate to the nucleus from the cytoplasm (Young et al., 2016). As mTORC1 is also negatively regulated by the AMPK-TSC2/raptor network (Kim et al., 2002). We detected this pathway-related protein expressions and found that PF11 did activate AMPK, leading to an increased phosphorylation of the two substrates p-TSC2 and p-Raptor leading to an inhibition of mTOR. Our data demonstrated that PF11 promotes nucleus translocation of TFEB in random-pattern skin flaps by modulating AMPK-mTOR signaling pathway.

Nevertheless, there are some limitations worth mentioning in this study. First, our results are based on *in vivo* experiments and no *in vitro* experiments were performed to determine other mechanisms by which PF11 enhanced the survival of the flaps. Second, compound C as a well-known AMPK inhibitor was used in this study. However, it is important to mention that a previous study screening for specificity of kinases inhibitors demonstrated that compound C can inhibit several kinases other than AMPK (Bain et al., 2007). Thus, it is not evident whether these kinases contribute to the nuclear translocation of TFEB promoted by PF11. Third, 3MA was used in this study to inhibited autophagy but 3MA does not directly destroy lysosomes and block autophagy. To better study the role of PF11 in autophagosome degradation, Chloroquine (disruption of lysosomal function inhibits autophagy) and bafilomycin A1 (inhibits the fusion of autophagosomes and lysosomes) should also be used. Moreover, whether there are other pathways involved in PF11-enhanced TFEB nuclear translocation requires further investigation. Notwithstanding these limitations, this study does suggest PF11 benefits for random-pattern skin flaps, widening the use range of PF11, and presents the benefits of PF11 and lays the foundation for further research.

## Conclusion

This study demonstrated that PF11 increases autophagy flux and then promotes angiogenesis, alleviates oxidative stress, and inhibits apoptosis in skin flaps. Moreover, we revealed that PF11 increases the nuclear translocation of TFEB via the AMPK-mTOR signaling pathway and ultimately induces autophagy. Overall, these results highlight the therapeutic benefits of PF11 in random-pattern skin flaps.

## Data Availability

The original contributions presented in the study are included in the article/Supplementary Material, further inquiries can be directed to the corresponding author.
